# Adsorptive and Electrochemical Properties of Carbon Nanotubes, Activated Carbon, and Graphene Oxide with Relatively Similar Specific Surface Area

**DOI:** 10.3390/ma14030496

**Published:** 2021-01-21

**Authors:** Krzysztof Kuśmierek, Andrzej Świątkowski, Katarzyna Skrzypczyńska, Lidia Dąbek

**Affiliations:** 1 Institute of Chemistry, Military University of Technology, 00-908 Warsaw, Poland; krzysztof.kusmierek@wat.edu.pl (K.K.); andrzej.swiatkowski@wat.edu.pl (A.Ś.); 2 Łukasiewicz—Industrial Chemistry Research Institute, 01-793 Warsaw, Poland; katarzyna.skrzypczynska@ichp.pl; 3 Faculty of Environmental, Geomatic and Energy Engineering, Kielce University of Technology, 25-314 Kielce, Poland

**Keywords:** single-walled carbon nanotubes, activated carbon, reduced graphene oxide, 2,4-D, adsorption, electroanalysis

## Abstract

Three carbon materials with a highly diversified structure and at the same time much less different porosity were selected for the study: single-walled carbon nanotubes, heat-treated activated carbon, and reduced graphene oxide. These materials were used for the adsorption of 2,4-D herbicide from aqueous solutions and in its electroanalytical determination. Both the detection of this type of contamination and its removal from the water are important environmental issues. It is important to identify which properties of carbon materials play a significant role. The specific surface area is the major factor. On the other hand, the presence of oxygen bound to the carbon surface in the case of contact with an organochlorine compound had a negative effect. The observed regularities concerned both adsorption and electroanalysis with the use of the carbon materials applied.

## 1. Introduction

The widespread use of chlorophenoxy herbicides in agriculture is an important source of soil and water contamination [1]. One of the most important herbicides, 2,4-dichlorophenoxyacetic acid (2,4-D), is used in several countries to control weeds. Even over 70 years after its introduction, 2,4-D continues to be the most common and widely used herbicide worldwide [2,3]. It is directly applied onto soil or sprayed over crop fields and, from there, often reaches surface waters and sediments. The choice of 2,4-D as the test substance was dictated by the fact that it belongs to a group of organochlorine pollutants to surface waters and groundwater which are characterized by high harmfulness of living organisms. The purpose of this work was to evaluate the adsorption potential of chosen carbon materials with a strongly differentiated structure for 2,4-D as the target water contaminant. So far, in research on the effectiveness of use as adsorbents against herbicides or phenol derivatives, activated carbons differing in porosity or surface chemistry were most often used [4,5,6,7]. Carbon materials with a different internal structure were used relatively rarely in such studies [8,9,10]. Moreover, many studies used carbon materials of various types with very different porosities (*S*_BET_) [8,9,10], which made it practically impossible to assess the influence of their internal structure differences on the adsorption efficiency.

Carbon materials, especially those classified into the graphite family, have a variety of textures (nanotexture and microtexture) and structures [11,12].

The graphite family refers to carbon materials that are similar to graphite in the sp^2^ hybridization of carbon atoms and the presence of several hexagonal carbon layers in the structure. There are numerous members of this family. For example, activated carbon that is formed by subjecting turbostratic carbon to a chemical reaction (activation) that consumed a part of the carbon. The activation results in surface pores, which cause the specific surface area to be high, as needed for fluid purification through the adsorption of the molecules to be removed.

The graphite family also includes graphite oxide, which is a covalent form of intercalated graphite. The conversion of graphite to graphite oxide involves its oxidation. The separation of the layers is a step in one of the methods of making graphene. The further reduction results in the removal of the oxygen and forming graphene oxide and, at the end, the reduced material known as reduced graphene oxide (rGO). The rGO sheets are usually considered as one kind of chemically derived graphene.

Carbon nanotubes (CNTs) are cylindrical molecules that consist of rolled-up sheets of single-layer carbon atoms (graphene). Among them, there are single-walled nanotubes (SWCNTs) with a diameter even less than 1 nm and multi-walled nanotubes (MWCNTs), consisting of several concentrically inter-linked nanotubes (diameters more than 100 nm). Like their building block graphene, CNTs are chemically bonded with sp^2^ bonds.

Carbon materials of a completely different type of structure were selected and used for the studies. The only common feature for them was a similar value of the specific surface area. The materials selected were commercial single-walled carbon nanotubes and reduced graphene oxide, as well as demineralized and high temperature, heated activated carbon to reduce its specific surface area.

In our work, apart from the adsorption properties of carbon materials, their electroanalytical usefulness was also tested. The electrochemical properties of carbon (graphite) paste electrodes (CPEs) modified by the addition of these carbon materials and their application in electroanalysis of 2,4-D were investigated.

## 2. Materials and Methods

### 2.1. Reagents and Materials

The graphite powder (45 μm), spectroscopic grade paraffin oil, as well as the 98% 2,4-dichlorophenoxyacetic acid (2,4-D), were purchased from Sigma Aldrich (St. Louis, MO, USA). Commercial, high-purity, open, single-walled carbon nanotubes (SWCNTs), from Nanostructured & Amorphous Materials, Inc. (Houston, TX, USA), were chosen for investigation. The second used carbonaceous nanomaterial was commercially reduced graphene oxide (rGO) received from Advanced Graphene Products Sp. z o.o. (Nowy Kisielin, Zielona Góra, Poland). The final carbon material used for comparison was extruded commercial activated carbon R3ex (Norit) demineralized with concentrated HF and HCl acids and next annealed in argon at 1800 °C (AC1800) [13]. All three carbon materials were selected in such a way that their specific surface area differs not too much. At the same time, their structure was extremely diverse.

### 2.2. Adsorbents Characterization

#### 2.2.1. Adsorption–Desorption N_2_ Isotherms

The porous structure of the carbon materials used for investigations was characterized using nitrogen adsorption–desorption isotherms at 77.4 K (ASAP 2020, Micromeritics, Norcross, GA, USA). On this basis, the main parameters characterizing the porosity of the chosen carbon materials, the specific surface area (*S*_BET_), micro- (*V*_mi_), and mesopore (*V*_me_) volumes were calculated.

#### 2.2.2. Microscopic Studies

Measurements of the surface-bonded oxygen content were carried out using a scanning microscope (Philips XL30/LaB6, Amsterdam, Netherlands) coupled with an energy dispersive X-ray spectrometer (DX4i/EDAX device). SEM images were also determined. High-resolution transmission electron microscopy (HRTEM) images for SWCNT were taken using a transmission electron microscope F20X-TWIN (FEI-Tecnai) operated at 200 kV [14].

#### 2.2.3. Raman Spectra

The characterization of the tested carbon materials was done by Raman spectroscopy using a Renishaw inVia Raman Microscope (Wotton-under-Edge, Gloucestershire, UK) with an exciting wavelength of 514.5 nm.

### 2.3. Adsorption from Aqueous Solutions

Batch experiments were carried out to examine the adsorption properties of the chosen carbon materials. All of the adsorption experiments were conducted at a room temperature of 25 °C in glassy Erlenmeyer flasks contacting different initial solutions of 2,4-D (0.02 L) with a given amount of carbon material (0.01 g).

The adsorption isotherms for the 2,4-D were constructed from solutions with an initial concentration ranging between 0.2 and 1 mmol/L. The flasks were then placed in the laboratory shaker and agitated at 100 rpm for 24 h. After this time, the samples were filtered through filter paper and analyzed for the 2,4-D content. The amount of the herbicide adsorbed per unit mass of adsorbent at equilibrium, *q_e_* (mmol/g), was calculated from the following formula:

(1) $$q_{e}=\frac{\left(C_{0}-C_{e}\right)V}{m}$$

where *C*_0_ and *C_e_* (mmol/L) are 2,4-D concentrations at initial and final steps, respectively, *m* (g) is the mass of the adsorbent added, and *V* (L) is the volume of the solution.

The kinetic studies were conducted for an initial 2,4-D concentration of 0.5 mmol/L. The flasks were agitated at 100 rpm. Samples were taken at different preset contact time intervals, filtered to prevent the presence of adsorbent in the samples, and analyzed spectrophotometrically. The amount of 2,4-D adsorbed at time *t*, *q_t_* (mmol/g), was evaluated by applying the following formula:

(2) $$q_{t}=\frac{\left(C_{0}-C_{t}\right)V}{m}$$

where *C_t_* (mmol/L) is the 2,4-D concentration at time *t*.

The quantification of the 2,4-D presents in aqueous solution was made using UV–Vis spectrophotometry (Carry 3E, Varian, Palo Alto, CA, USA) at the wavelength of 278 nm. The calibration curve was constructed (0.02–0.8 mmol/L) by plotting absorbances vs. 2,4-D concentrations (y = 0.956x + 0.035; R² = 0.999).

### 2.4. Voltammetry

All the voltammetric measurements were conducted with an AutoLab PGSTAT 20 (Eco Chemie, Utrecht, Netherlands) potentiostat. For all electrochemical measurements, the conventional three-electrode system was used: (1) a modified carbon paste electrode, (2) a Pt wire, and (3) a saturated calomel electrode, which functioned as the working electrode, the auxiliary electrode, and the reference electrode, respectively. Differential pulse voltammetry (DPV) was used in this study. The measurements were carried out in a homemade 40 mL glass cell. Voltammograms were registered from 0 to 2.0 V at a sweep rate of 50 mV/s. The pulse height and width were set as 50 mV and 50 ms, respectively, and the sampling time was 50 ms. A Teflon holder with a hole at one end for filling the carbon paste served as the electrode body. Electrical contact was made with a stainless-steel rod through the center of the holder. Modified CPEs were prepared by thoroughly mixing 2.5, 5, or 10 wt.% of modifying carbon material and graphite powder with the subsequent addition of mineral oil. All ingredients were placed in an agate mortar, crushed with a pestle and the mixture was kept at room temperature for 3 days. The prepared paste was then packed into the hole of the electrode body and smoothed onto a paper.

## 3. Results and Discussion

### 3.1. Physicochemical Characterization of the Carbon Materials

**Figure 1:**
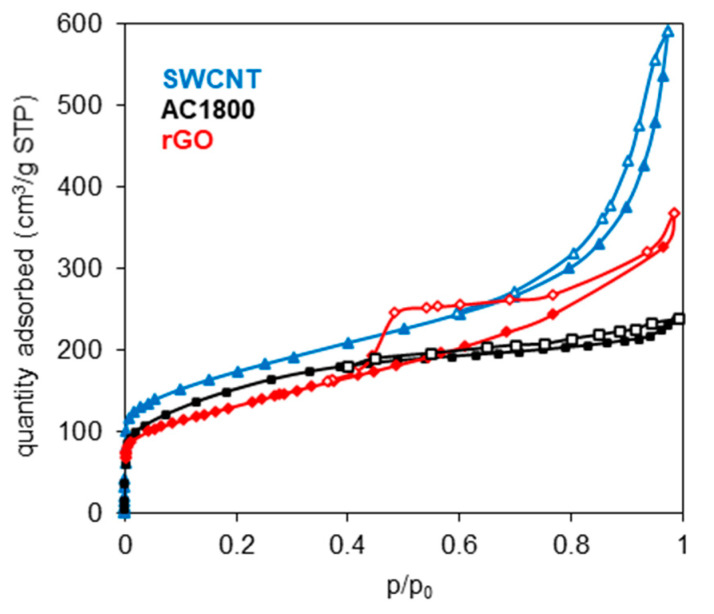
Nitrogen adsorption–desorption isotherms (measured at 77 K) of single-walled carbon nanotubes (SWCNTs), AC1800, and rGO.

The low-temperature nitrogen adsorption–desorption isotherms on the carbon materials are presented in Figure 1. The specific surface areas (*S*_BET_), as well as the micropore (*V*_mi_) and mesopore (*V*_me_) volumes, were calculated from the N_2_ adsorption isotherms, and the results are listed in Table 1. The *S*_BET_ was calculated by the BET method, while the *V*_mi_ and *V*_me_ volumes were calculated using the t-plot method.

Table 1 shows that the specific surface areas of the tested materials are not very diverse (the maximum difference between them is 85 m^2^/g). A similar relationship can be noticed in the case of the micropore volume, while there are significant differences in the mesopore volume—the AC1800 exhibits the lowest *V*_me_ as a result of its high-temperature treatment [7,13].

**Figure 2:**
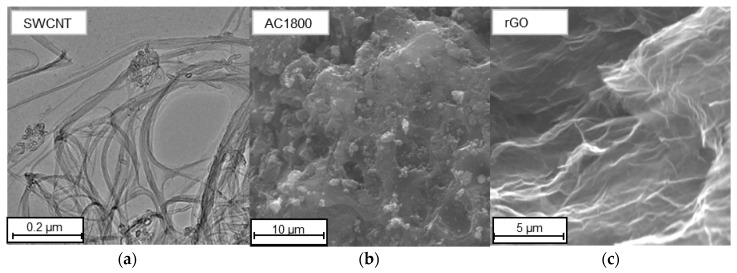
The TEM (SWCNT) [14] and SEM images of the carbon materials. (**a**) SWCNT; (**b**) AC1800; (**c**) rGO.

The results of the performed HRTEM analysis for SWCNT [14] are presented in Figure 2a–c. As can be observed, CNTs are long and partially opened. On the external walls, some amorphous carbon forming the debris is observed. The images (Figure 2) show a large variation in the surface morphology of the tested carbon materials, resulting from their internal structure.

**Figure 3:**
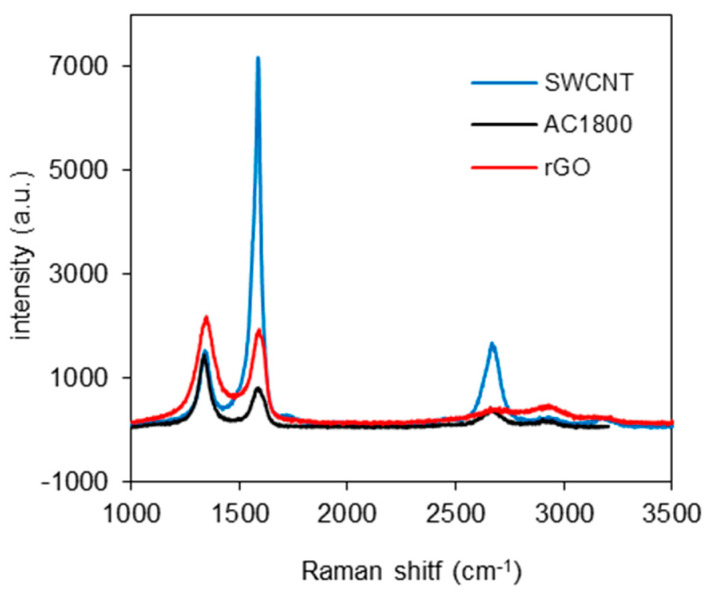
The Raman spectra of the SWCNT, AC1800, and rGO.

Figure 3 shows the Raman spectra obtained for the tested carbon materials. The peaks at about 1350 and 1580 cm^−1^ correspond to D and G bands, respectively. The D-band represents the A_1g_ vibration mode caused by the disordered structure of the carbon materials, whereas the G-band corresponds to the E_2g_ vibration mode in the graphitic lattice of carbon materials [15].

For the carbon materials used in this research, the D-band was located at 1348, 1350, and 1349 cm^−1^ for SWCNT, AC1800, and rGO, respectively, and the G-band was at 1573, 1581, and 1588 cm^−1^, respectively. Using the ratio of peak intensities I_D_/I_G_, one can use Raman spectra to characterize the disorder level in carbon materials. The appropriate values were 0.031, 2.21, and 1.30 for SWCNT, AC1800, and rGO, respectively. As one can see, the carbon materials tested are highly differentiated in terms of internal structure arrangement. SWCNT shows the highest difference in intensities of the D and G bands [14,16]. The opposite situation can be observed for the heat-treated activated carbon AC1800. The heating temperature (1800 °C), although significantly reducing its pore volume, is too low for graphitization [13].

The relative intensity ratio of both peaks (I_D_/I_G_) for the rGO sample is a measure of disorder degree and is inversely proportional to the average size and number of the sp^2^ clusters [15,17].

### 3.2. Adsorption Study

The adsorption of the 2,4-D from aqueous solutions onto single-walled carbon nanotubes, heat-treated activated carbon, and reduced graphene oxide was studied by means of the adsorption kinetics and the construction of adsorption isotherms.

**Figure 4:**
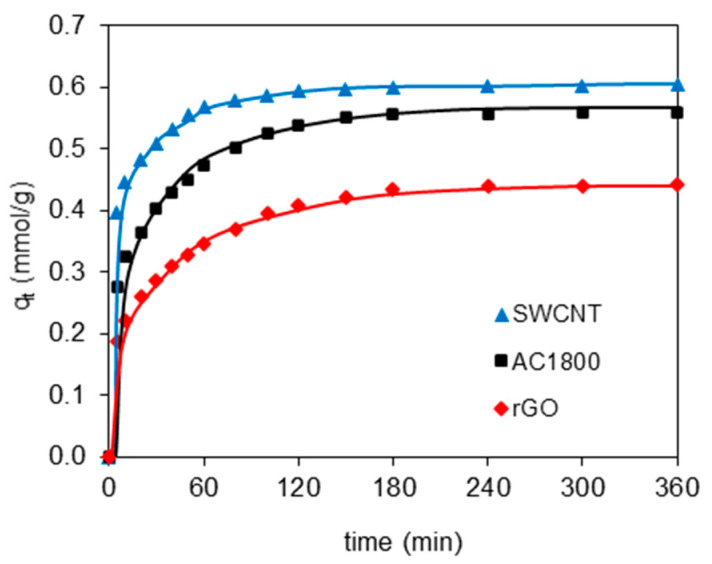
The effect of contact time on 2,4-D adsorption onto SWCNT, AC1800, and rGO.

Adsorption kinetics of 2,4-D from the water on the carbonaceous materials is shown in Figure 4. It was observed that the adsorption rapidly increased in the first steps of the process and reached the adsorption equilibrium within about 1–2 h.

To further analyze the adsorption kinetics, the data from Figure 4 were fitted by pseudo-first-order (PFO) (Equation (3)) and pseudo-second-order (PSO) (Equation (4)) kinetic models:

(3) $$\log\left(q_{e}-q_{t}\right)=\log q_{e}-\frac{k_{1}}{2.303}t$$

(4) $$\frac{t}{q_{t}}=\frac{1}{k_{2}q_{e}^{2}}+\frac{1}{q_{e}}t$$

where *k*_1_ and *k*_2_ are the rate constants of PFO (1/min) and PSO adsorption (g/mmol·min), respectively.

The values of *k*_1_ and *k*_2_ were calculated from the slope and intercept of the plots of log(*q_e_* − *q_t_*) versus *t* and *t*/*q_t_* versus *t*, respectively, and are given in Table 2.

The R^2^ values for the PSO kinetic model are equal or greater than 0.999 for adsorption of the herbicide on all of the carbonaceous materials. Furthermore, a better agreement between the experimental (*q_e_*_(EXP)_) and calculated (*q*_e(CAL)_) values of equilibrium adsorption capacity was observed for the PSO kinetic model than for the PFO. This suggests that the adsorption of the 2,4-D on the adsorbents follows the pseudo-second-order kinetic model.

The adsorption results revealed that the 2,4-D was adsorbed faster on the SWCNT than on the rGO and that the microporous material of heat-treated activated carbon led to a much longer time to reach the adsorption equilibrium. The values of the *k*_2_ for 2,4-D followed the sequence: AC1800 < rGO < SWCNT. This order in the rate of adsorption can be explained by the different porous structures of the materials—the content of mesopores, which play the role of transporting arteries. The adsorption rate increased with an increase in the content of mesopores in the total porous structure of the carbon materials.

**Figure 5:**
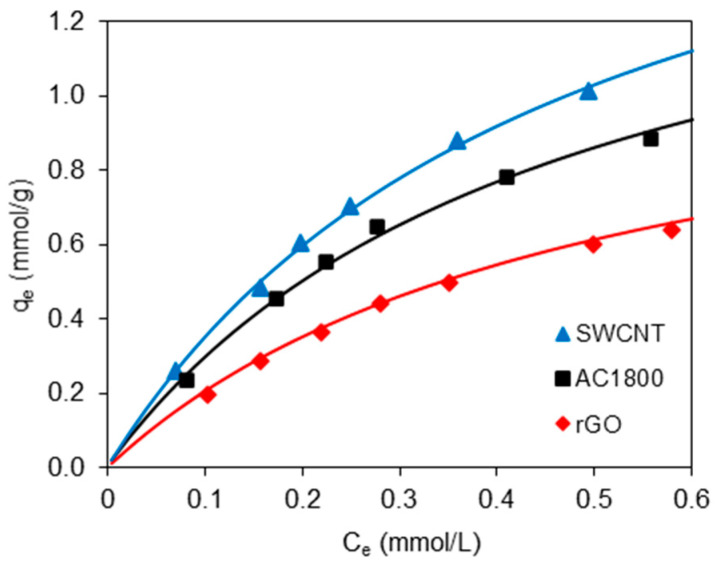
Adsorption isotherms of 2,4-D on SWCNT, AC1800, and rGO.

Adsorption isotherms of the 2,4-D on the SWCNT, AC1800, and rGO are presented in Figure 5. To understand the adsorption isotherm, the data from Figure 5 were fitted by the Langmuir (Equation (5)) and Freundlich (Equation (6)) models with the following nonlinear forms:

(5) $$q_{e}=\frac{q_{m}bC_{e}}{1+bC_{e}}$$

(6) $$q_{e}=K_{F}C_{e}^{1/n}$$

where *q_m_* (mmol/g) is the maximum adsorption capacity, *b* (L/mmol) is the Langmuir parameter, while the *K_F_* ((mmol/g)·(L/mmol)^1/*n*^) and *n* are the Freundlich constants.

The suitability of these models was verified based on the correlation coefficient R^2^ and standard deviation equation Δ*q* (%) as:

(7) $$\Delta q=100\times \sqrt{\frac{\Sigma {\left[\left(q_{exp}-q_{cal}\right)/q_{exp}\right]}^{2}}{N-1}}$$

The results (Table 3) show that the Langmuir and Freundlich isotherm models fitted reasonably well the adsorption data for all of the adsorbents. However, the Langmuir model fitting was slightly better for experimental data due to the higher R^2^ and lower Δ*q* values. This suggested the monolayer and homogeneous adsorption of 2,4-D onto the SWCNT, AC1800, and rGO surface.

Equilibrium adsorption experiments revealed that the SWCNT had the highest 2,4-D adsorption capacity, compared to the AC1800 and rGO adsorption capacities. These findings can be attributed to the SWCNT’s high specific surface area and micropore volume values that facilitate the efficient adsorption of 2,4-D. Both, the Langmuir and Freundlich parameters (*q_m_* and *K_F_*) follow the order of the BET specific surface areas (rGO < AC1800 < SWCNT). After converting the adsorption per 1 m^2^ of surface area, a relatively small difference between SWCNT and AC1800 (0.0033 and 0.0030 mmol/m^2^, respectively) can be observed, and in the case of rGO, this value (0.0024 mmol/m^2^) was much lower than for the two previous materials.

The worst adsorption capacity of the rGO can be explained by taking into account one more factor besides *S*_BET_, namely, the surface chemistry, and more specifically the amount of oxygen bound to the carbon surface. While in the case of SWCNT and AC1800, it was relatively small and comparable, in the case of rGO, it was very large (several times greater). The presence of the acidic surface oxygen groups decreases the adsorption efficiency. This phenomenon is associated with the hydration of polar carboxyl groups leading to the creation of water clusters, which can block active sites on the adsorbent surface and reduce its availability for adsorbent molecules [6,18]. In many studies, it was found that the presence of oxygen on the surface of carbon materials reduces the adsorption of organic compounds of similar structure to 2,4-D, e.g., 2-(4-chloro-2-methylphenoxy)acetic acid (MCPA), 2-(4-chloro-2-methylphenoxy)propanoic acid (MCPP), and 4-(4-chloro-2-methylphenoxy) butanoic acid (MCPB) [6].

Comparisons of 2,4-D monolayer adsorption capacity of the SWCNT, AC1800, and rGO with those of other adsorbents reported in previous studies are given in Table 4. The adsorption capacities reported in this study are high compared to other carbonaceous materials.

### 3.3. Electroanalytical Research

**Figure 6:**
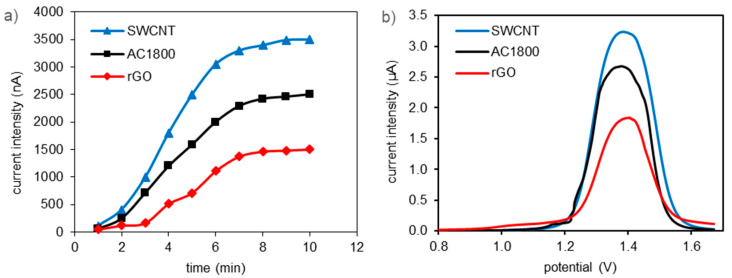
Effect of accumulation time on peak current for 0.5 mmol/L 2,4-D (**a**); differential pulse voltammetry (DPV) registered for 0.5 mmol/L 2,4-D solutions using carbon paste electrodes (CPEs) containing 10% by mass of the tested materials (**b**).

The first stage of DPV studies was the evaluation of the effect of the accumulation time on the peak current of the oxidation of 2,4-D in a solution (0.5 mmol/L) for the CPEs modified with all the tested carbon materials. The current intensity was found to increase with the accumulation time until 7 min, after which it maintained a constant value (Figure 6a). In further studies, an accumulation time of 7 min was used.

Voltammograms for the CPE as an example with 10% content of carbon modifiers are shown in Figure 6b. From all the DPV curves (without as well as with 2.5%, 5%, and 10% modifiers content), the peak currents and the peak potentials were determined.

Similar to the dependence of the amount of adsorbed 2,4-D (*q_m_*) on the value of the specific surface area (*S*_BET_) of the carbon material, changes of the peak currents can be observed, recorded during the voltammetric measurements with the use of a carbon paste electrode modified by the addition of SWCNT, AC1800 or rGO. The values of the peak currents decrease with the decrease in the specific surface area of the CPE modifiers for different amounts of their additions and different concentrations of 2,4-D solutions. Similar relationships have already been described in the literature [7,8]. They have even been shown to be linear [8]. In our case, the decreasing relationship is also close to linear, but the peak current for CPE modified by the addition of rGO shows too much decrease.

For example, for the concentration of the 2,4-D solution 0.5 mmol/g and the content of the CPE modifiers 10% wt., the peak intensity (μA)/S_BET_ (m^2^/g) ratios for SWCNT and AC1800 are 0.00552 and 0.00538, respectively, while for rGO it is only 0.00431. This too low value for rGO as a CPE modifier results, as can be considered, from the high oxygen content in this carbon material.

The differential pulse voltammetry was applied to determine the concentration of 2,4-D using the modified CPEs in the range of 0.002–0.5 mmol/L. Calibration curves were fitted by linear regression using the peak current versus concentration and the detection (LOD) and quantitation (LOQ) limits of the CPEs were calculated using the following formulas:

(8) $$\mathrm{LOD}=\;\frac{3\sigma }{a}$$

(9) $$\mathrm{LOQ}=\;\frac{6\sigma }{a}$$

where *a* is a slope of the calibration curve (y = ax + b), and *σ* is a standard deviation of the blank signal.

The linear regression equations, as well as the respective correlation coefficients, are presented in Table 5.

The sensitivity of all modified electrodes was much greater than that of the unmodified (graphite) electrode. Moreover, the sensitivity of the methods was correlated with the amount and type of modifier. The LOD decreased with the increase of the amount of the modifier content from 2.5 to 10% as well as with the *S*_BET_ of the carbon materials used. The best sensitivity (0.468 μmol/L) was observed for the CPE modified with SWCNT (10%). The sensitivity of these methods is comparable to other electrochemical methods for 2,4-D determination described elsewhere. The LOD was found to be 0.08 μmol/L for graphite-polyurethane electrode [26], 0.2 μmol/L for R3ex activated carbon modified CPE [8], 0.23 μmol/L for mercury electrode [27], 0.400 µmol/L for silica-gel modified CPE [28], 0.7 μmol/L for Carboxen 1000 carbon molecular sieve modified CPE [8], 0.83 μmol/L for electrochemical sensor based on molecularly imprinted polypyrrole membranes [29], 0.98 μmol/L for F-300 activated carbon modified CPE [23], 1.14 μmol/L for SX-2 activated carbon modified CPE [23], 3.15 µmol/L for bismuth film modified screen-printed carbon electrode [30], and 3.4 μmol/L for Carbopack B carbon black modified CPE [8].

## 4. Conclusions

The conducted research has shown that the adsorption and electrochemical properties of carbon materials in solutions of aromatic organochlorine compounds depend on, to a large extent, their porosity. In the case of the presence of a larger amount of oxygen bonded with their surface than usual (only a few % by weight), a worsening of their adsorption and electrochemical properties can be observed. The general conclusions are as follows:

- The higher the specific surface, the better the adsorption, as well as electroanalytical properties, of carbon materials;
- Suitability of carbon materials for adsorption and electroanalysis seems to be correlated;
- The internal structure of carbon materials affects their surface characteristics;
- In the case of organochlorine compounds, the presence of oxygen on the carbon surface reduces their adsorption.

## Figures and Tables

**Table 1:** Physicochemical properties of the carbon materials tested.

Adsorbent	*S*_BET_ (m^2^/g)	*V*_mi_ (cm^3^/g)	*V*_me_ (cm^3^/g)	*V*_mi_/*V*_mi_ + *V*_me_	Oxygen Surface Content, (% wt.)
SWCNT	597	0.267	0.314	0.460	4.9
AC1800	554	0.239	0.133	0.642	1.4
rGO	512	0.220	0.272	0.447	17.1

**Table 2:** The pseudo-first- and pseudo-second-order rate constants for adsorption of 2,4-D on the carbonaceous materials.

Adsorbent		Pseudo-First-Order			Pseudo-Second-Order		
	*q_e_*_(EXP)_ (mmol/g)	*k*_1_ (1/min)	R^2^	*q_e_*_(CAL)_ (mmol/g)	*k*_2_ (g/mmol∙min)	R^2^	*q_e_*_(CAL)_ (mmol/g)
SWCNT	0.604	0.022	0.935	0.423	0.049	0.999	0.612
AC1800	0.559	0.019	0.975	0.323	0.016	0.999	0.579
rGO	0.441	0.021	0.992	0.305	0.038	0.999	0.462

**Table 3:** The Langmuir and Freundlich isotherm equation parameters for adsorption of 2,4-D on the carbon materials.

Adsorption Model	Parameter	SWCNT	AC1800	rGO
Langmuir	*q_m_* (mmol/g)	2.001	1.652	1.222
	*b* (L/mmol)	2.127	2.168	2.030
	R^2^	0.991	0.993	0.994
	Δ*q* (%)	2.139	2.888	4.244
Freundlich	*K_F_* ((mmol/g)·(L/mmol)^1/*n*^)	1.806	1.462	0.934
	*n*	1.403	1.439	1.540
	R^2^	0.977	0.975	0.972
	Δ*q* (%)	5.229	7.462	5.231

**Table 4:** Comparison of the 2,4-D adsorption on various adsorbents.

Adsorbent	*S*_BET_ (m^2^/g)	Langmuir Adsorption Capacity, *q_m_* (mg/g)	Ref.
SWCNT	597	442.3	this paper
AC1800	554	365.1	this paper
rGO	512	270.1	this paper
groundnut shell char	43	3.02	[19]
CB-C carbon black	97	68.6	[20]
CB-V carbon black	227	72.2	[20]
AC from sugarcane bagasse	507	153.9	[21]
AC from groundnut shell	709	250.0	[19]
commercial AC F-400	800	137.7	[22]
commercial AC SX-2	885	180.4	[23]
AC from coconut shell	991	233.0	[21]
commercial AC F-300	965	191.2	[23]
AC from data stones	763	238.0	[24]
AC from corncob	1274	300.0	[25]

**Table 5:** Linearity results for the modified carbon paste electrode.

Electrode	Linear Regression Equation	R^2^	LOD	LOQ
	y = ax + b		(µmol/L)	(µmol/L)
graphite CPE	y = 0.054x + 0.006	0.992	50	100
SWCNT (2.5%) CPE	y = 3.484x + 0.076	0.999	0.775	1.549
SWCNT (5.0%) CPE	y = 4.931x + 0.109	0.997	0.555	1.095
SWCNT (10%) CPE	y = 5.760x + 0.138	0.998	0.468	0.937
AC1800 (2.5%) CPE	y = 3.111x + 0.015	0.992	0.868	1.736
AC1800 (5.0%) CPE	y = 4.833x + 0.089	0.994	0.561	1.117
AC1800 (10%) CPE	y = 5.378x + 0.171	0.995	0.502	1.004
rGO (2.5%) CPE	y = 2.480x − 0.029	0.997	1.089	2.177
rGO (5.0%) CPE	y = 3.631x + 0.074	0.995	0.744	1.487
rGO (10%) CPE	y = 4.061x + 0.161	0.996	0.673	1.330

## Data Availability

Data sharing is not applicable to this article.
